# Toward Pristine Return: Probabilistic Sample Response Modeling of Vibration Sensing for Planetary Samples

**DOI:** 10.3390/s26175618

**Published:** 2026-09-04

**Authors:** Caitlin Ahrens

**Affiliations:** 1 Center of Research and Exploration in Space Science and Technology II (CRESST-II), University of Maryland, College Park, MD 20742, USA; cahrens@umd.edu; 2 NASA Goddard Space Flight Center, Greenbelt, MD 20771, USA

**Keywords:** sample return, sample integrity, sample technology, vibration sensors

## Abstract

**Highlights:**

**What are the main findings?**

- Various planetary mission phases may impose different levels of vibration on a collected sample.
- Future lunar volatile samples could face mechanical changes from the containment and transport processes if not monitored properly.

**What are the implications of the main findings?**

- Dedicated vibration sensors should be integrated into a planetary sample container design for sample knowledge and curation practices.
- Sample handling of lunar polar volatiles should involve vibration monitoring, and any potential scientific loss should be back-modeled.

**Abstract:**

Planetary sample return missions can impose extreme mechanical forces on both spacecraft and the returned samples collected from a planetary surface that they contain. The chain of custody phases from entry/descent/landing to curation each present distinct vibration, shock, and dynamic loads that may compromise sample integrity. Interplanetary sample return missions, from the Apollo program to Stardust, Hayabusa, Hayabusa2, and OSIRIS-REx (Origins, Spectral Interpretation, Resource Identification, and Security—Regolith Explorer), have refined capsule design to ensure sample integrity. While much attention has understandably been paid to contamination control, thermal control, and mechanical shock at impact, the role of vibrational monitoring on the sample container itself is less frequently explored; yet, it merits attention. Here, we combine a review of vibration sensor aspects relevant to planetary sample return with a Monte Carlo analysis of how capsule-level vibration may propagate into representative lunar sample types. Using Apollo-derived sample container geometries and a representative multi-frequency capsule vibration environment, we show that sample archetypes can exhibit substantially different dynamic responses to the same vibration input. These results illustrate the value of vibration measurements at or near the sample container for documenting the mechanical environment experienced by returned material and interpretation of potential scientific losses during transport and curation.

## 1. Introduction and Heritage

Sample collection and coring have been the foundation of many exploratory investigations of the Earth, from investigating geologic evolution and deep time to providing mineral resources and geotechnical foundations for mining and manufacturing. Vibrational sensors play a key role in monitoring the mechanical integrity and environmental history of materials. These measurements are particularly important for unconsolidated sediments, fractured rock, hydrate-bearing formations, and other mechanically fragile materials, where dynamic excitation may induce particle rearrangement, fracture propagation, pore collapse, or changes in fluid distribution. Vibration-specific sensors would record the amplitude, frequency spectrum and time history of the mechanical loads that are experienced, enabling scientists and curators to (1) identify whether the sample was exposed to unexpected shock or vibration that might induce fracturing, microcracks, or redistribution of chemicals and volatiles; (2) correlate specific dynamic events with possible changes in sample structural integrity (e.g., loss of layering, deformation of shrink swell features, bubble collapse); and (3) support decisions in curation workflows (such as prioritizing which subsamples may need non-destructive evaluation first, or which may need special handling/storage). Consequently, vibration sensing (at least on Earth) has evolved from a diagnostic tool for drilling systems into a means of documenting sample provenance and preservation throughout the chain of custody. In geotechnical, petroleum, marine sediment, and cryogenic ice core applications, vibration measurements are used to quantify shock events, characterize drill dynamics, assess handling-induced disturbances, and provide objective evidence that recovered samples have remained within acceptable mechanical exposure limits.

Insight into the evolution of planetary surfaces (both ancient and recent processes) and the physical and chemical materials thereof can be gained by directly sampling and collecting samples from those planetary bodies. The National Academies Planetary Science and Astrobiology Decadal Survey 2023–2032 (2023) [1], the Artemis III Science Definition Team Report [2], and reports supporting the Mars Sample Return [3,4] each have specific objectives for the importance of and need to collect samples. Recently, the Lunar Exploration Analysis Group (LEAG) and Extraterrestrial Materials Assessment Group (ExMAG) have also led a recent special action team (SAT) to define knowledge gaps regarding frozen volatile sample collection from the Moon, including recommendations about their curation [5]. While many planetary discussions focus on thermal shielding and biological contamination concerns [6,7], the mechanical loading history (vibration, shock, acceleration, rotational motion) experienced by a sample return capsule (SRC) can critically influence sample preservation (i.e., through cracking, degassing, or shifting of particulate layers).

A targeted literature search was conducted using Semantic Scholar, covering publications from the preceding 10 years. Searches (at the time of writing this manuscript) included “container vibration monitoring” (38,600 results); “vibration planetary sample contamination” (692 results); “lunar sample return” (148 results); “vibration sensor for sample return” (19 results); “vibration sensor for ice cores” (9 results); and “planetary sample return capsule container vibration monitoring” (5 results). Publications were considered based on their relevance to planetary sample return vibration environments, vibration and shock sensing, sample containment, contamination or sample disturbance, and technologies applicable to monitoring mechanical environments experienced by returned samples or planetary surface sample analyses. Relevant mission-specific literature and reports were also considered where appropriate. The search was intended as a targeted review rather than a systematic review with formal screening and meta-analysis.

**Figure 1:**
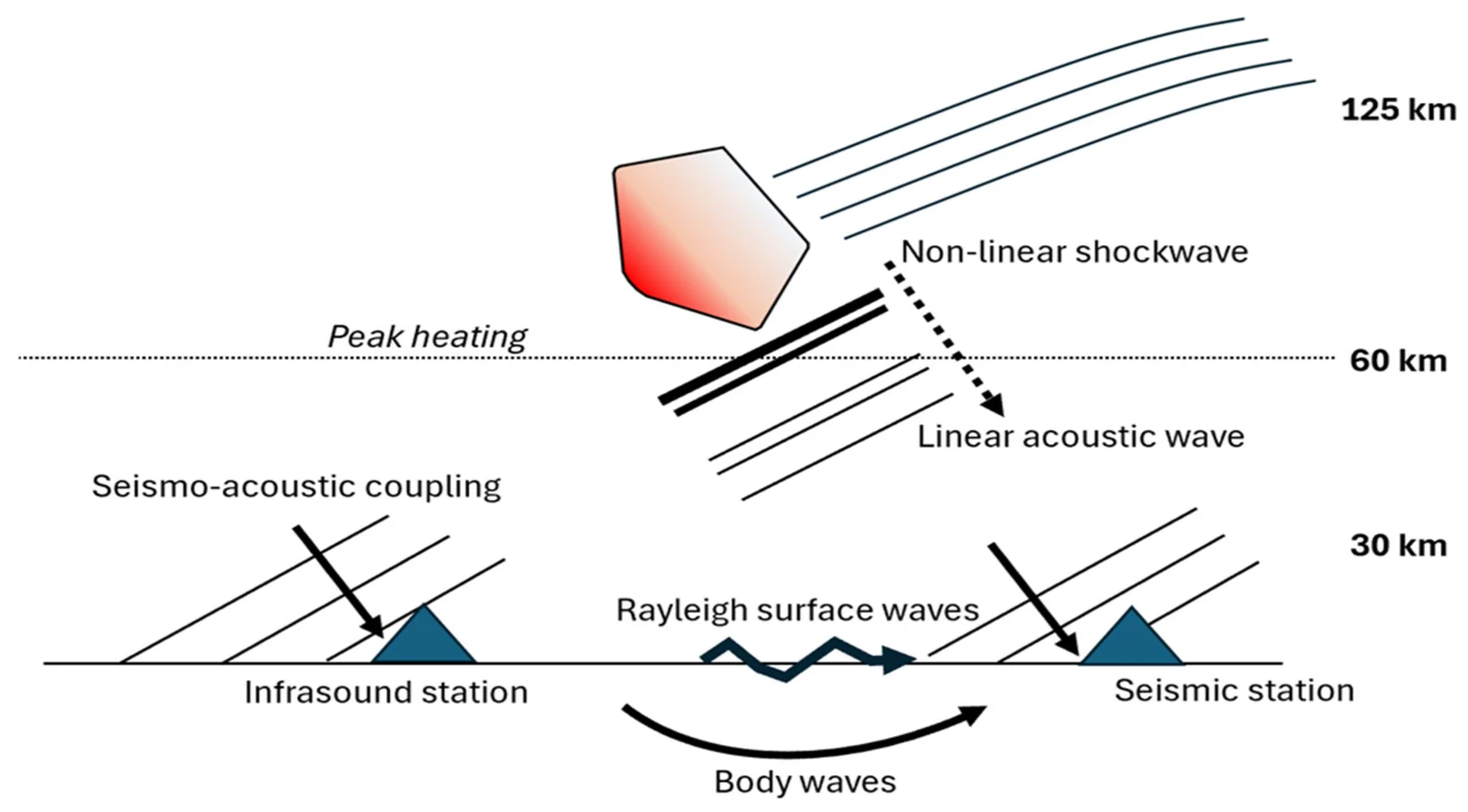
Schematic representation (*not to scale*) of shockwave generation during reentry of a sample return capsule. Illustration adapted from [10,11]. Note the variety of shockwave environments that a return capsule must endure, highlighting the need for advanced sample container monitoring.

Engineering aspects focused on ensuring sample safety are prioritized during the return of the capsule [8,9]. However, the reentry is mainly focused on the safety of the capsule itself, and trajectories and retrievals are planned well in advance. Since the Apollo era, only a limited number of planetary sample return objects, specifically five, have been documented using ground-based geophysical instrumentation, providing a range of velocity, trajectory, and capsule descent maneuvers. Although the capsules themselves are often sparsely instrumented (and therefore sparsely recorded), the placement and geometry of reconnaissance instruments and sample container handling are of the utmost importance. Recently, sensing and ground-based seismic instrumentation have been used as the capsule interacts with Earth’s atmosphere (Figure 1) to assess shockwave seismic dynamics.

Although vibration monitoring is not always foregrounded in sample return mission reports, the evidence suggests that mission engineers are aware of dynamic loads and their implications for sample integrity. Yet there remains a gap, in that dedicated vibrational sensing at the sample container during all mission phases, especially beyond EDL into surface operations, storage, transport, and curation, is rarely documented in the literature. This gap justifies the argument for a structured investment in vibrational monitoring strategies.

Unlike terrestrial coring operations, current planetary sample return missions provide no opportunity to inspect or re-acquire specimens if mechanical disturbance occurs after collection. Consequently, a vibration sensing system integrated directly into the sample container can function as a scientific witness sensor, continuously documenting the dynamic mechanical environment experienced by the sample from acquisition through laboratory curation. Such a system would generate an objective record of acceleration, shock, and vibration exposure that accompanies the returned specimen as part of its metadata. Rather than simply verifying spacecraft performance, the sensor data would provide quantitative evidence regarding the mechanical history of the sample itself, allowing investigators to assess whether observed microstructural, mineralogical, or volatile characteristics may have been altered by transport-induced disturbances. This report reviews the heritage of previous planetary sample return capsules equipped with complementary seismic suites and vibration-specific sensor technologies for terrestrial coring and sampling and discusses the need for vibration sensors for future lunar volatile sample return.

## 2. Previous Return Capsules

Although previous ground-based seismic arrays measure the external propagation of shockwaves of a planetary sample return capsule interacting with the atmosphere, rather than internal stresses, their records contain the magnitude, frequency magnitude, and duration of vibrational events that the spacecraft experiences. However, they do not necessarily quantify the vibration transmitted to the sample container or the sample itself. Distinguishing these measurement locations is important because the dynamic response of the contained material depends on its mechanical properties and on the frequency content of the imposed vibration. The following examples therefore distinguish between ground-based observations and spacecraft or capsule measurements.

### 2.1. Apollo

The 382 kg of lunar material returned by the six Apollo missions (Apollo 11, 12, 14, 15, 16, 17) remain foundational to our understanding of planetary science, but their impact extends well beyond pure research. Each Apollo mission had dedicated microphones and seismic arrays for launch and landing, which led to investigating overall flight integrity and sonic speed measurements during reentry [12,13]. For stakeholders, including commercial entities, national space agencies, and international partnerships, these legacy samples serve as critical benchmarks for developing return technologies, contamination control protocols, and sample curation standards. Moreover, these samples continue to generate new insights decades later, highlighting the long-term scientific value of carefully preserved extraterrestrial materials, such as the ANGSA (Apollo Next Generation Sample Analysis) program.

### 2.2. Genesis and Stardust

The Genesis sample return capsule crash-landed in the Utah desert on 8 September 2004, after both its drogue parachute and parafoil failed to deploy, causing it to impact the ground at approximately 310 km/h [14]. Despite severe damage, including a crushed section of the cylindrical sample container, scientists salvaged valuable solar wind samples. The unique materials used for the collectors, such as sapphire and diamond (resistant to scratching), aided in the recovery process.

The Stardust sample return capsule (SSRC) returned to Earth on 15 January 2006. It was designed to collect and return samples from comet 81P/Wild2, as well as interstellar dust. The SSRC had an initial entry velocity of 12.5 km/s, which was the highest ever achieved by an artificial object entering Earth’s atmosphere [15]. Various broadband photometric, spectrographic, infrasonic, optical, and seismic instruments were deployed to record the Stardust SSRC reentry, including a collocated infrasound seismic array setup in Wendover, Utah.

### 2.3. Hayabusa1 and Hayabusa2

Hayabusa1, despite technical setbacks, successfully returned the first asteroid samples from the asteroid Itokawa, proving the feasibility of autonomous rendezvous, sampling, and return from a near-Earth object. Acoustic and infrasonic waves were observed during reentry of the Hayabusa asteroid SRC at six ground sites in Woomera, Australia on 13 June 2010 [16]. The spacecraft’s main bus fragmented at 75–38 km of altitude, with a few observed explosive emissions. Based on video evidence from multiple ground sites, approximately 136 fragmented parts of the spacecraft were analyzed and classified as explosive, melting, or re-fragmented [16].

Hayabusa2 built upon this foundation with refined navigation and redundant systems and made multiple sampling attempts at asteroid Ryugu, including subsurface material retrieval via a kinetic impactor. The Hayabusa1 and Hayabusa2 capsule designs enabled passive thermal protection and minimal atmospheric alteration upon Earth reentry, providing valuable data on surface–atmosphere interactions.

### 2.4. OSIRIS-REx

Returning material from the asteroid Bennu, the OSIRIS-Rex (Origins, Spectral Interpretation, Resource Identification, and Security—Regolith Explorer) mission delivered the largest carbon-rich asteroid sample to date (~122 g), offering critical insights into the building blocks of the early solar system and potential prebiotic chemistry [17]. Unlike previous missions, OSIRIS-REx also emphasized meticulous sample handling, with multiple layers of containment to protect sample integrity from terrestrial contamination and atmospheric alteration during reentry. What sets OSIRIS-REx apart for future-oriented mission planning is the opportunity it presented to pair sample return with a dedicated seismic monitoring array near the Utah Test and Training Range (UTTR), where the capsule landed [18]. A miniature inertial measurement unit (IMU) developed by Honeywell Aerospace was used for accelerometry during cruise, reentry, parachute deployment, and impact [19]. Table 1 summarizes each of these historic and recent planetary sample return mission sensors, noting that terrestrial seismic and acoustic-type sensors have been the most used thus far, but that there is a lack of container-specific sensors and collection monitoring.

These examples demonstrate that mechanical environments associated with sample return missions have been characterized using a range of ground-based, spacecraft-level, and external sensing approaches. However, these measurements do not necessarily quantify the mechanical environment experienced directly by the sample container or its contents.

## 3. Terrestrial Coring Sample Systems

Most terrestrial coring systems employ microelectromechanical system (MEMS) accelerometers [20]. High-resolution three-axis MEMS accelerometers are frequently integrated into autonomous data loggers that record acceleration, peak shock, vibration spectra, and event-triggered time histories. In drilling environments characterized by severe impacts or stick-slip behavior, piezoresistive MEMS accelerometers are often selected because they tolerate substantially higher overload conditions while maintaining adequate frequency response. For very high-frequency measurements, such as drill bit dynamics and piston-related impact characterization, piezoelectric accelerometers remain widely used because they exhibit excellent sensitivity and can withstand harsh thermal and mechanical environments. In petroleum and mineral exploration, downhole vibration monitoring routinely combines tri-axial accelerometers with gyroscopes and magnetometers to form IMUs [21]. Ultimately, sensor selection should be based on the expected mechanical environment, measurement location, and required sensitivity rather than a fixed set of specifications. For the present conceptual analysis, the sensor technology itself is not prescribed; instead, the analysis focuses on the mechanical response that a suitably selected container-level sensor could characterize.

Ice cores on Earth illustrate how mechanical loads, transport stress, and internal structural fragility interact to compromise scientific outcomes. In one example, the RICE (Roosevelt Island Climate Evolution) project ice core team observed that, below about 475 m in depth, the ice became “too brittle to cut without significant damage” and mitigated this by buffering the sections in a refrigerated snow cave for ~14 days prior to shipment, thereby reducing breakage in transport. Another example is the WAIS (West Antarctic Ice Sheet) Divide ice core project, where the core quality in the brittle zone (~650 m to ~1300 m) degraded significantly, with spontaneous fracturing soon after recovery; the team netted the cores to contain fragments and limit damage during handling [22].

In many cases, vibration records are archived alongside metadata describing temperature history, orientation, pressure, and environmental conditions, thereby providing a comprehensive record of sample custody. This approach has become increasingly important as geochemical, microbiological, and microstructural analyses require confidence that observed features are representative of the in situ environment rather than artifacts introduced during recovery or transport.

## 4. Future Lunar Ice Samples

Damage to (presumed) frozen volatile lunar samples collected from the permanently shadowed regions (PSRs) at the lunar poles poses serious risks to the scientific objectives of future sample return missions, especially given the current uncertainty surrounding the physical nature, composition, and microstructure of these volatiles (i.e., porosity, grain size, crystal habit, tensile strength). Unlike terrestrial ice and polar ice cores, lunar ice exists in an extremely cold (typically <100 K) [23], airless, and chemically reactive environment, potentially as a mixture of water ice, exotic volatiles (e.g., CO_2_, SO_2_, NH_3_, CH_4_), and solar wind-induced molecules adsorption to regolith grains at varying depths. Disruption during sampling, transport, or reentry could irreversibly alter this complex volatile inventory and bring about some degree of scientific loss.

First, mechanical damage such as fracturing, vibration-induced sublimation, or loss of pressure containment could lead to partial or total degassing of volatile compounds. Since the isotopic composition of these volatiles is crucial for identifying their origins (e.g., cometary impacts, solar wind implantation, volcanism) [24,25], even small alterations in composition caused by sample disturbance may compromise the ability to distinguish between competing formation hypotheses.

Second, the physical state of lunar volatiles is poorly constrained [26]. Frozen volatiles may exist as discrete ice grains, surface frosts, hydrated minerals, amorphous coatings, or even nanophase adsorbates on regolith particles. Each of these forms has distinct thermal and mechanical sensitivities. Without knowing the thermal and chemical regime of these forms, it is difficult to predict what mechanical thresholds could be safely tolerated for a “pristine” sample.

Third, microstratigraphy may be critical. Layering within PSR regolith might preserve episodic deposition of volatiles. Vibrational damage could disturb this layering, mixing volatile-bearing horizons with sterile material, thereby obscuring their temporal and spatial resolution. This is especially concerning given the small mass of material likely to be recovered.

Granular materials such as lunar and asteroid regolith are highly sensitive to repeated dynamic loading, even at relatively low acceleration amplitudes. Cyclic vibration can induce particle rearrangement, densification, stratigraphic disturbance, and size segregation through well-established granular physics mechanisms, including kinetic sieving and granular convection [27]. One of the best-known manifestations is the Brazilian nut effect, in which repeated vibration causes larger particles or clasts to migrate relative to finer grains due to collective particle motion rather than simple gravitational settling.

Rasera et al. [27] demonstrated that vibrational segregation of Apollo lunar samples and relevant granular simulants provides an effective “dry” alternative for analyzing particle size distributions, particularly for fine and cohesive materials where fluid-based characterization methods are impractical. These findings are relevant to ISRU, as accurate characterization of particle size distributions can improve the handling and processing of feedstock materials for additive manufacturing on planetary surfaces and support the efficient extraction of oxygen and valuable metals from regolith. While the reduced gravity environment of the Moon, Mars, or asteroids modifies the rate and magnitude of these processes, laboratory experiments and numerical discrete element method (DEM) simulations have demonstrated that vibration-induced segregation remains active under reduced gravitational acceleration, although the governing thresholds and timescales differ from terrestrial conditions.

## 5. Monitoring Across Mission Phases

**Figure 2:**
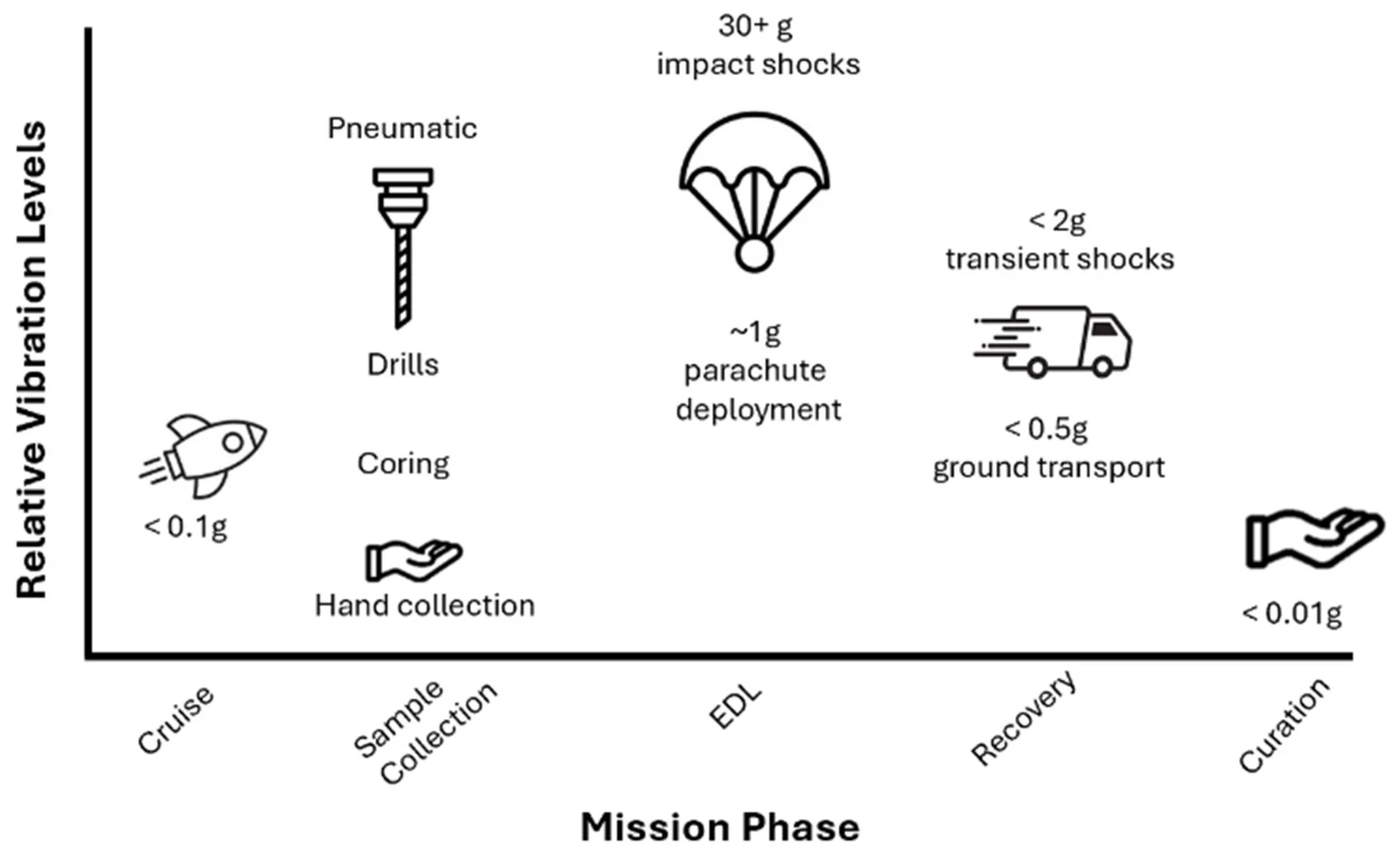
Mission phase components with approximate and representative vibrational processes. Typical vibration magnitudes are shown in terms of peak acceleration (where 1 g = 9.8 m s^−2^). Exact vibration intensity values vary depending on vehicle and sample container, as these values are purely illustrative.

Effective vibrational monitoring for planetary sample return requires an integrated approach that spans all mission phases, from launch to curation, because each stage presents distinct mechanical environments and potential threats to sample integrity (Figure 2). Despite decades of spacecraft qualification testing and environmental modeling, there remains a critical knowledge gap in quantifying the actual vibrational environment experienced by the sample container and its contents during flight and post-landing operations.

The cruise and transfer phases introduce (relatively lower magnitude) vibration. Reaction wheel desaturation, attitude control maneuvers, docking operations, and internal mechanism actuation all impart sustained low-frequency vibrations. Historically, these have been considered inconsequential, yet cumulative or resonant effects may alter the structure of fine-grained or weakly consolidated samples.

Pneumatic sampling systems, like those demonstrated on OSIRIS-REx, generate high-frequency pulses from gas flow and valve cycling, while rotary drilling induces complex torque and impact vibrations that risk altering fine-scale sedimentary structures. Compared to passive coring, these methods impose elevated mechanical loads at the point of contact with the sample. Peak acceleration identifies isolated shock events capable of inducing brittle fracture or particle displacement, whereas acceleration quantifies sustained vibration exposure during rover traverses or spacecraft propulsion maneuvers. Techniques like frequency domain analysis using fast Fourier transform (FFT) or power spectral density (PSD) estimation identify dominant excitation frequencies that may coincide with resonant modes of the sample container or internal regolith.

The EDL sequence represents the most severe dynamic phase. Data from missions such as Stardust, Hayabusa1 and Hayabusa2, and OSIRIS-REx have demonstrated the feasibility of high-fidelity monitoring of EDL events using onboard accelerometers and external sensing campaigns. While these efforts have greatly advanced understanding of atmospheric entry dynamics, translating this heritage into container-level monitoring remains an open opportunity. Integrating miniature accelerometers into the container or its mounting structure could bridge this gap, enabling correlation between vehicle dynamics and the mechanical stresses potentially transmitted to the sample itself.

Following touchdown, the recovery and transport phases introduce additional vibrational exposures that have received comparatively little study. Surface impact, retrieval operations, and terrestrial transport to curation facilities can subject the container to shock and low-frequency vibration that may go unrecorded. Real-time or data-logging sensors embedded in the container could capture these environments, allowing curation teams to assess whether any mechanical disturbances occurred after landing. Such information would be especially valuable for volatile- or ice-rich samples, where even minor agitation may induce structural or chemical changes.

Finally, during curation and long-term storage, vibration monitoring can ensure that laboratory environments maintain the stability necessary for sample preservation. Handling, storage equipment, and environmental control systems may impart small but cumulative vibrations, particularly in cryogenic or vacuum-preserved contexts. Continuous or event-triggered sensing can verify that mechanical conditions remain within allowable limits throughout curation and analysis, completing a continuous mechanical history from collection to study.

## 6. Return Capsule Structural Integrity

While monitoring the sample within the capsule is useful, monitoring the capsule integrity itself can also be beneficial. The size, geometric structure (internally and externally), and material nature of the return capsules can vary from mission to mission. These data can be leveraged to optimize crucial aspects such as the capsule’s design, landing strategies, and sample handling procedures.

**Figure 3:**
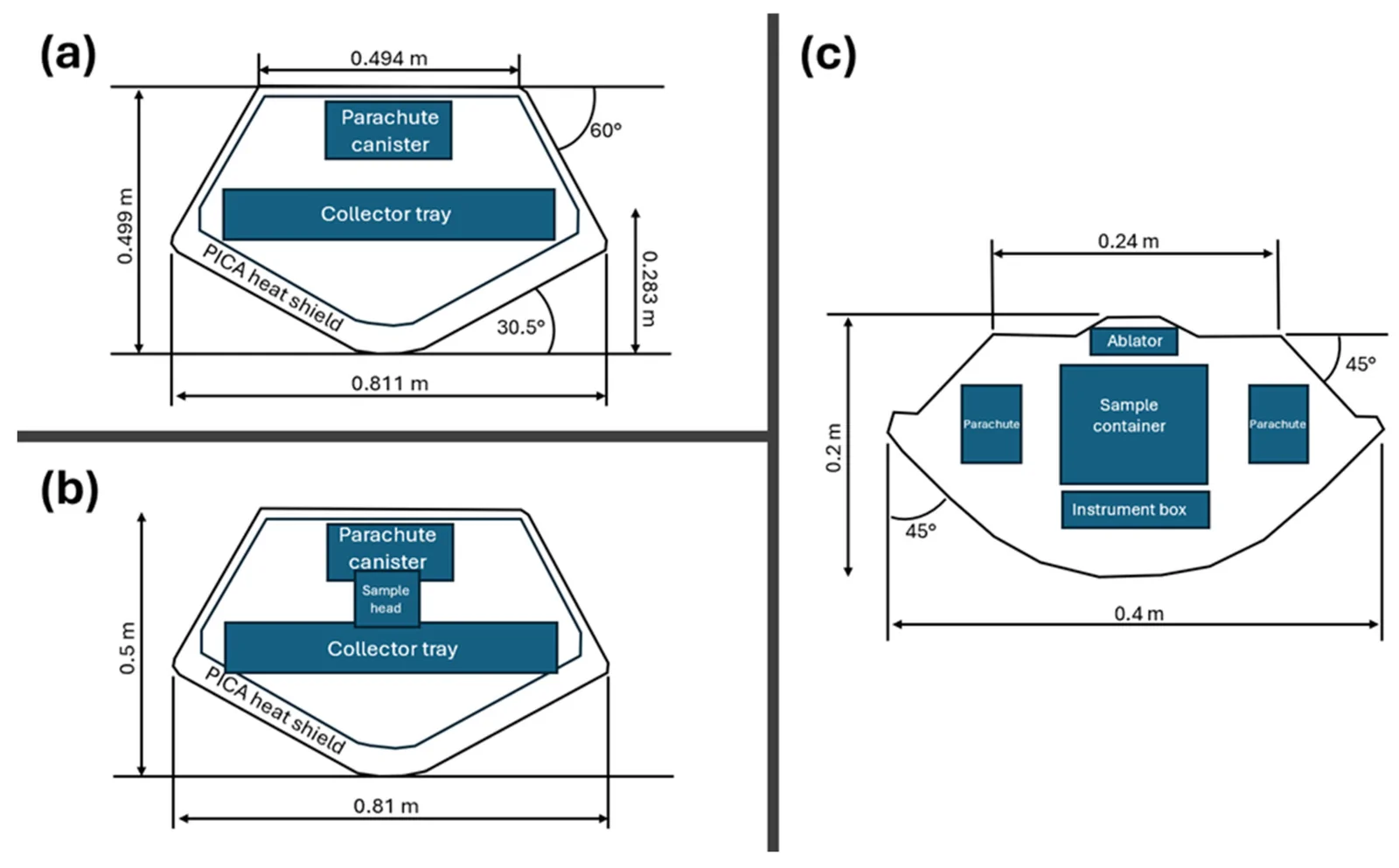
Comparative SRC schematics from previous missions illustrating the internal placement and geometry of collection containers and necessary protective equipment. (**a**) Stardust [28]; (**b**) OSIRIS-REx [29]; (**c**) Hayabusa [30].

To ensure structural integrity, the capsule design must be robust enough to endure the dynamic pressures and aerodynamic loads encountered during reentry (Figure 3). Factors such as acceleration, vibration, and potential collisions with micrometeoroids or space debris must be considered. Maintaining the capsule’s structural robustness is crucial to preventing it from breaking apart or becoming deformed, which could jeopardize the integrity of the sample container. Looking at previous capsule designs in Figure 3, we note that the collector tray (or sample container) is well within the center of the capsule, typically built on a rigid platform, and a vibration sensor could indeed be placed at the base and top of the tray for sample monitoring purposes. This is also important for when parachutes deploy, which would be considered a very short but intense burst of vibration.

## 7. Sample Integrity and Contamination

The Stardust and Genesis missions underscore the challenges of preserving sample integrity during unexpected mechanical events, including hard landings and environmental exposure [31,32]. For missions governed by stringent planetary protection protocols, such as Category V restricted Earth returns (e.g., Mars sample return), monitoring and timestamping mechanical disturbances post-landing is particularly valuable [4]. These protocols aim to prevent both forward contamination of other planetary bodies and backward contamination of Earth with potentially hazardous materials.

From a curation perspective, vibrational data contextualize the condition of re-turned materials, enabling facilities to assess whether samples experienced damaging shocks or resonance effects that could induce fracturing, phase changes, or chemical and volatile redistribution. These metadata not only inform tailored storage strategies and advancing capabilities regarding non-destructive testing, but also promote reproducibility and scientific transparency, allowing future researchers to account for transport-induced artifacts in compositional or textural analyses. Over time, accumulated datasets will facilitate comparative studies across missions and contribute to establishing best practices in planetary sample transport and curation.

Taken together, the preceding examples demonstrate that vibration and shock environments can be characterized at several points along the sample return chain, but measurements at the spacecraft or capsule level do not necessarily establish the mechanical environment experienced by the contained sample. This distinction is particularly important for lunar materials whose mechanical properties, packing states, and dynamic responses may vary substantially among sample types. To explore the potential consequences of this uncertainty, we developed a probabilistic analytical framework that propagates uncertainty in representative lunar sample properties through their dynamic response to a prescribed capsule vibration environment.

## 8. Probabilistic Vibration Analysis of Lunar Sample Types

### 8.1. Representative Lunar Sample Archetypes

To examine how uncertainty in sample properties may influence the mechanical response of returned material, three representative lunar sample archetypes were defined: bulk regolith, fine dust, and volatile-bearing regolith. These archetypes were selected to represent distinct physical states that could plausibly be encountered in future lunar sample return missions, while avoiding the implication that they correspond to three formally defined lunar sample classes.

The bulk regolith archetype represents a relatively heterogeneous granular sample dominated by the mechanically coupled behavior of typical lunar regolith. The fine dust archetype represents a more finely divided material with potentially different packing and effective mechanical properties. The volatile-bearing regolith archetype represents regolith containing volatile-bearing phases (or deposits) and is included as a conceptual archetype, because pristine lunar volatile samples have not yet been returned and experimentally characterized under representative transport conditions.

The distinctions among these archetypes are motivated by the known variability of lunar regolith physical and mechanical properties. Previous Apollo measurements demonstrate substantial variation in bulk density with sampling location and depth, while studies of lunar regolith and simulants have documented variations in grain size, packing, porosity, and cohesion behavior. These variations provide a physical basis for treating sample mechanical properties as uncertain rather than assigning a single deterministic response.

For the analysis, each archetype was assigned a representative mass and associated distributions of mechanical properties. The modeled sample mass assumption for all sample archetypes was 15.0 g. This mass is a modeling assumption intended to provide comparable representative sample quantities and is not intended to represent measured masses of future returned lunar samples.

The volatile-bearing archetype is therefore treated explicitly as a hypothetical modeling category. Its purpose is to explore whether a sample with different assumed mechanical properties could exhibit a different dynamic response in the same imposed vibration environment. The analysis does not assume that vibration produces volatile loss, sublimation, phase change, or redistribution; those processes require separate experimental and physical validation.

### 8.2. Apollo-Derived Sample Container Geometry

To provide a physically grounded basis for the analytical framework, sample container geometries were derived from Apollo-era sample return hardware. Two geometries were considered: the Apollo Lunar Sample Return Container (ALSRC) and the Apollo 17 drive tube associated with sample 73001. These containers provide representative examples of lunar sample containment across substantially different scales and geometries.

The modeled ALSRC has a calculated internal volume of 24,119.445 cm^3^. For the present analysis, a nominal 50% fill fraction was used to define a representative sample volume of 12,059.7225 cm^3^. The modeled 73001 drive tube has a calculated internal volume of 414.690 cm^3^ and was treated as fully filled, giving a representative sample volume of 414.690 cm^3^. These geometric calculations are used to characterize the physical scale of the containment systems and provide a consistent basis for subsequent calculations. The analysis does not attempt to reproduce the mass loading of a specific Apollo sample container; instead, it uses Apollo hardware to provide a realistic geometric context for investigating how uncertainty in representative sample properties may influence vibration response.

### 8.3. Mechanical Properties and Uncertainty

The dynamic response of a granular sample contained within a return capsule depends on its effective mechanical properties, including density, stiffness, and damping. These properties are expected to vary with particle size and composition, packing state, porosity, and cohesion. Consequently, assigning a single deterministic value to each property would not adequately represent the uncertainty associated with returned lunar materials, particularly for sample types that have not yet been directly characterized under representative transport conditions.

For the present analysis, uncertainty was therefore introduced into the sample density, effective stiffness, and damping properties using probability distributions. The distributions were applied independently within the Monte Carlo framework to generate a range of plausible mechanical states for each sample archetype. The resulting realizations were then propagated through the natural frequency and vibration transmissibility calculations.

The three archetypes were assigned different representative mechanical property distributions to reflect their conceptual physical differences. Bulk regolith was treated as a relatively heterogeneous granular material, fine dust as a more finely divided material with potentially different packing and effective stiffness, and volatile-bearing regolith as a distinct conceptual material state for which direct mechanical characterization under sample return conditions is not currently available.

The adopted property ranges (Table 2) should therefore be interpreted as modeling assumptions rather than direct experimental measurements of pristine lunar samples. In particular, the volatile-bearing archetype is intended to explore the consequences of mechanical property uncertainty rather than to reproduce a quantitatively validated material model for lunar volatile deposits. The Monte Carlo analysis consequently does not provide a measurement of the mechanical properties of returned lunar samples; instead, it demonstrates how uncertainty in those properties can propagate into uncertainty in predicted vibrational dynamic response.

For each realization, the sample natural frequency was calculated from the effective stiffness and sample mass according to

(1) $$f_{n}=\frac{1}{2\pi }\sqrt{\frac{k_{eff}}{m}},$$

where $f_{n}$ is the natural frequency, $k_{eff}$ is the effective stiffness, and m is the nominal 15 g sample mass. The resulting distribution of natural frequencies provides the basis for calculating frequency-dependent transmissibility and sample acceleration in the representative capsule vibration environment described below.

### 8.4. Representative Hypothetical Capsule Vibration Environment

**Figure 4:**
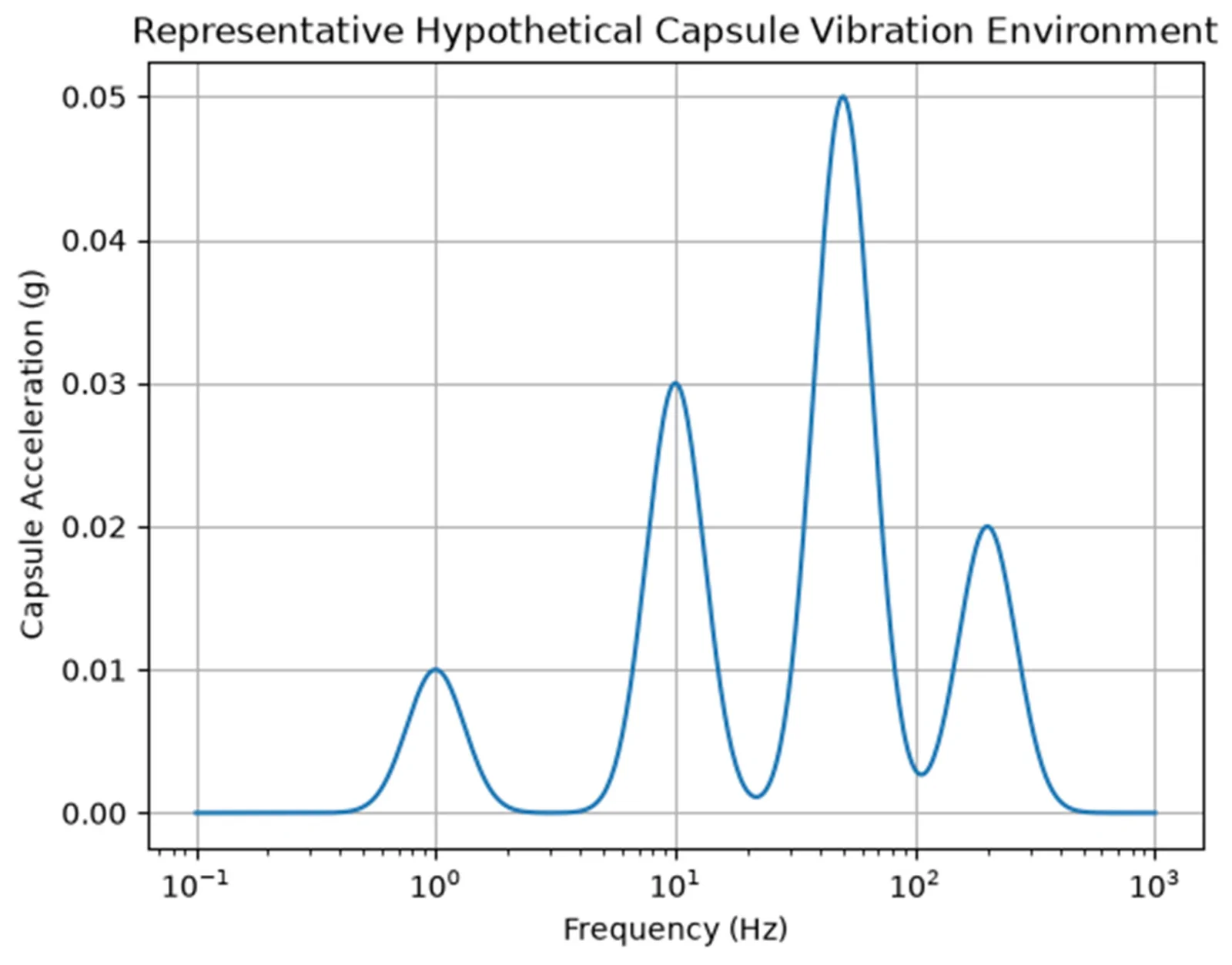
Illustrative hypothetical capsule vibration environment used as the excitation input for the Monte Carlo analysis. Four discrete excitation components at 1, 10, 50, and 200 Hz were used to examine frequency-dependent sample response.

The representative capsule vibration environment was constructed as a smooth frequency-dependent spectrum containing the four excitation components. A baseline acceleration level of approximately 0.01 g was used for the input environment, with the individual frequency components providing localized increases in excitation amplitude. The resulting spectrum is shown in Figure 4.

The selected frequencies span low-, intermediate-, and higher-frequency portions of the modeled response and were selected to interrogate different portions of the natural frequency distributions produced by the model. The 1 and 10 Hz components represent relatively low-frequency excitation, while the 50 and 200 Hz components provide excitation near and above portions of the natural frequency distributions obtained for the three sample archetypes. This allows the same imposed vibration environment to be evaluated against samples with different characteristic dynamic responses.

The hypothetical nature of the input environment is important to the interpretation of the results. The purpose of this analysis is not to estimate the vibration environment of a particular mission, landing system, or transport scenario, but to demonstrate the analytical relationship between capsule-level vibration and potential sample-level response. A future mission-specific implementation could replace the representative spectrum with measured or experimentally validated vibration data from launch, spacecraft operations, EDL, recovery, or transport.

### 8.5. Monte Carlo Natural Frequency Analysis

The propagation of mechanical property uncertainty was evaluated using a Monte Carlo simulation consisting of 10,000 realizations for each sample archetype. For each realization, the density-adjusted sample mass, effective stiffness, and damping ratio were sampled from the distributions described in Section 8.3. The resulting natural frequency was then calculated for each realization using the single-degree-of-freedom approximation described above.

A common vibration environment does not necessarily produce a common response within different sample materials, because the relationship between imposed vibration frequency and sample response depends on the mechanical properties of the material. Thus, measurement of the vibration environment at the capsule or container level provides information about the imposed excitation, but interpretation of the mechanical response of the contained sample requires consideration of sample-specific properties.

To evaluate how differences in sample mechanical properties may influence response to capsule vibration, a representative hypothetical vibration environment was constructed. The environment was designed to contain multiple excitation frequencies spanning the frequency range over which the modeled sample archetypes exhibited substantial variation in natural frequency.

**Figure 5:**
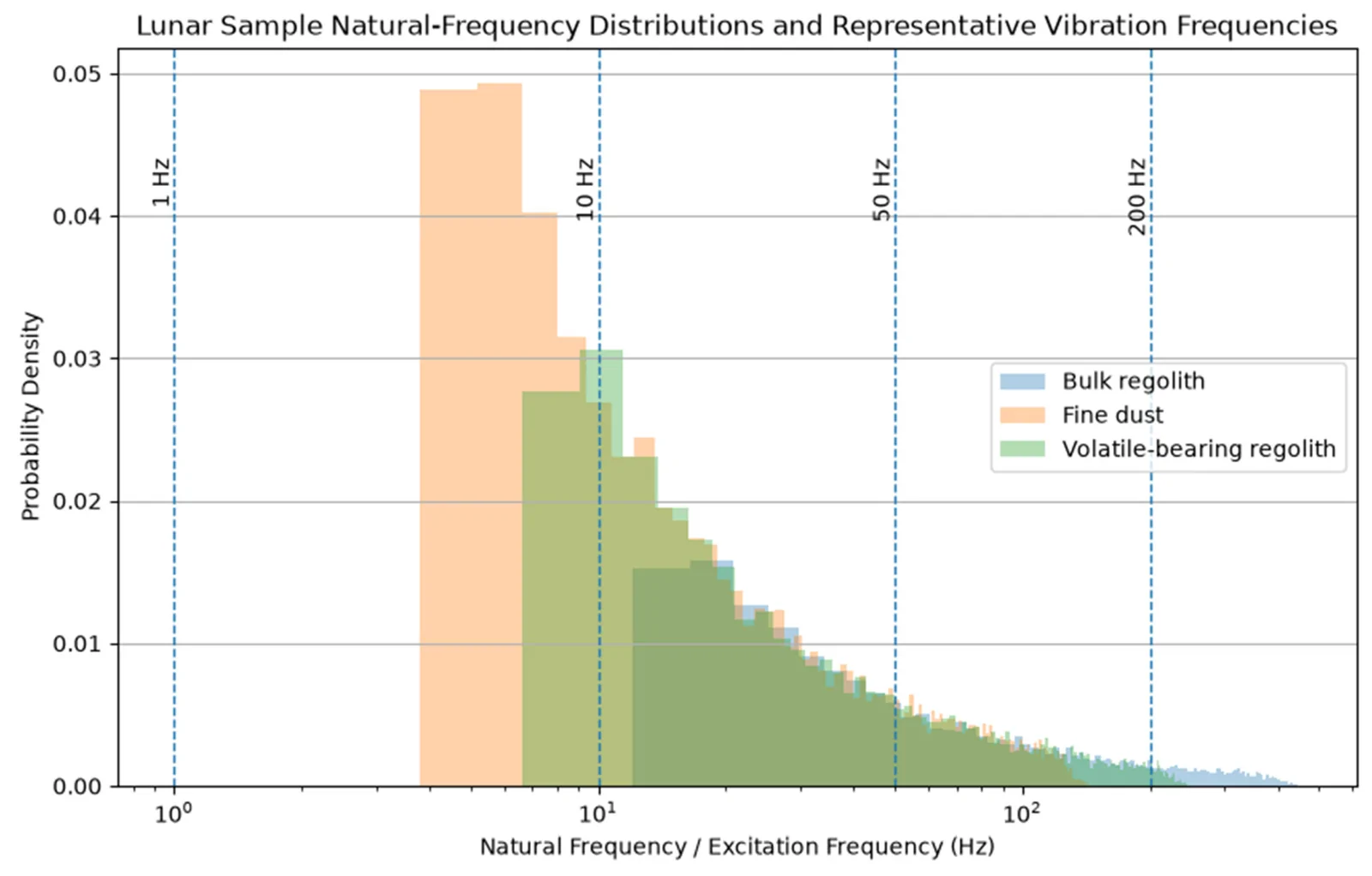
Monte Carlo-derived natural frequency distributions for the three representative lunar sample archetypes and the four excitation frequencies used in the hypothetical capsule vibration environment.

The resulting natural frequency distributions differed substantially among the three sample archetypes (Figure 5). The bulk regolith archetype produced a median natural frequency of 73.4 Hz, with a 5th–95th percentile range of approximately 15.5–344.8 Hz. The fine dust archetype exhibited a lower median natural frequency of 24.9 Hz and a 5th–95th percentile range of approximately 5.2–114.3 Hz. The volatile-bearing regolith archetype produced an intermediate median natural frequency of 39.7 Hz, with a 5th–95th percentile range of approximately 8.5–189.3 Hz.

These results demonstrate that the assumed differences in sample mechanical properties can produce substantially different dynamic characteristics even when the nominal sample mass is held constant. In particular, the fine dust archetype exhibits a lower characteristic natural frequency and a broader distribution extending into the low-frequency range, while the bulk regolith archetype is characterized by a higher median natural frequency and a distribution extending to substantially higher frequencies. The volatile-bearing archetype falls between these two cases.

These frequencies were not intended to reproduce a measured vibration spectrum from a specific lunar sample return mission. Rather, they provide a simplified excitation environment for demonstrating how a common capsule-level vibration input can produce different responses in materials with different dynamic characteristics. The use of discrete frequency components also permits the relationship among excitation frequency, sample natural frequency, and vibration transmissibility to be examined directly.

The Monte Carlo results should be interpreted as a demonstration of this sensitivity rather than as predictions of the natural frequencies of specific returned lunar samples. The distributions arise from the assumed parameter ranges and therefore illustrate the potential consequences of mechanical property uncertainty in the absence of direct experimental characterization.

### 8.6. Propagation of Capsule Vibration into Sample Response

The representative capsule vibration environment was propagated through the dynamic response model for each of the three lunar sample archetypes. For each Monte Carlo realization, the frequency-dependent transmissibility was calculated from the sampled natural frequency and damping ratio, and the resulting sample acceleration was obtained from the imposed capsule acceleration and corresponding transmissibility.

**Figure 6:**
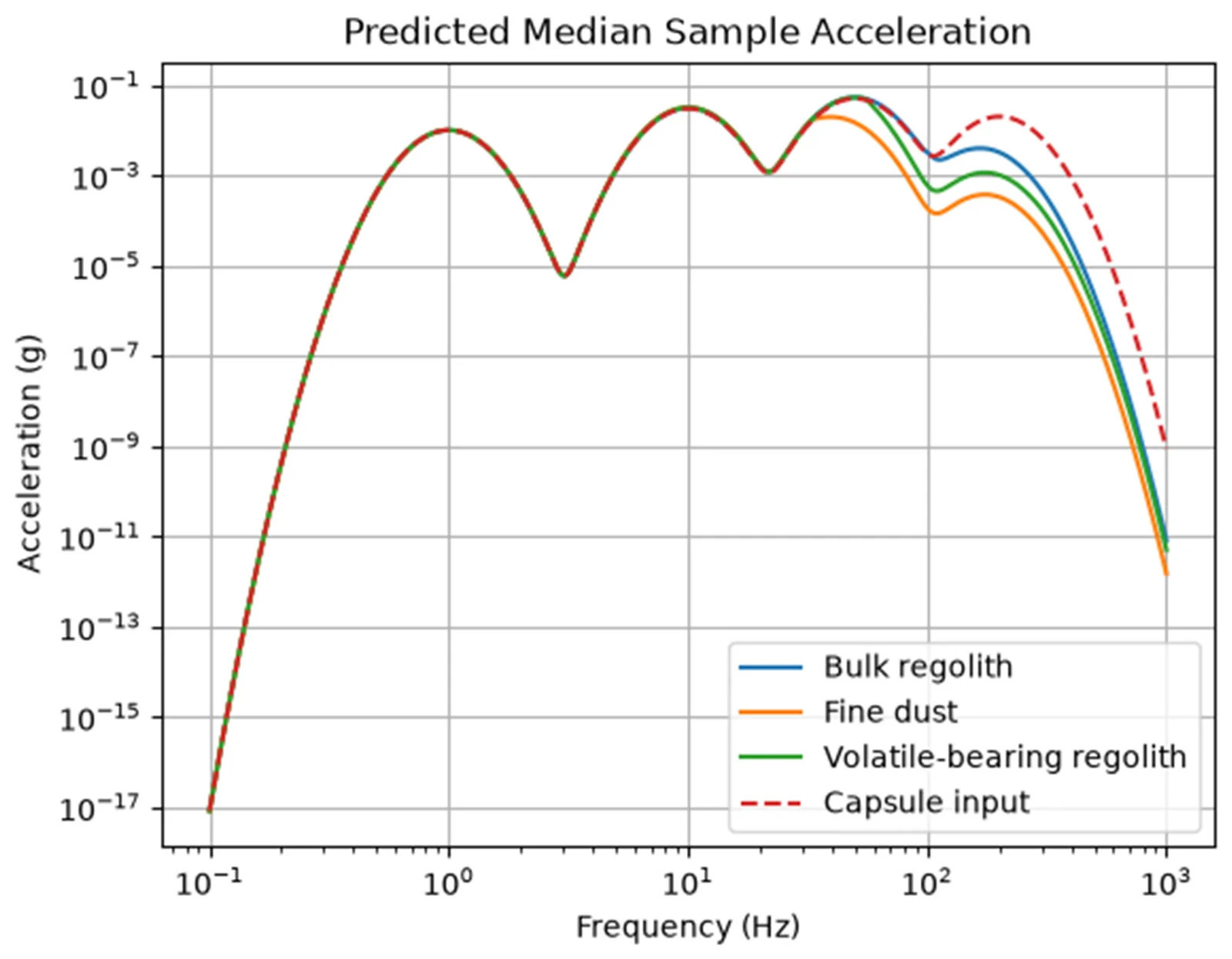
Predicted median sample acceleration as a function of vibration frequency for the three representative lunar sample archetypes compared with the representative capsule vibration environment. The archetype responses remain relatively similar at low frequencies but progressively diverge at >35 Hz, reflecting differences in their assumed mechanical properties and frequency-dependent transmissibility.

The predicted median sample acceleration responses are shown in Figure 6. At low frequencies, the responses of the three archetypes remain relatively similar and approach the imposed capsule acceleration. Greater separation occurs at higher frequencies, where the differences in natural frequency distributions and damping characteristics produce increasingly different transmissibility responses.

The modeled response exhibits peaks near the four excitation frequencies of the representative capsule environment. However, the magnitude of these responses differs among the sample archetypes. The bulk regolith archetype generally produces the largest response among the three archetypes over portions of the higher-frequency range, followed by volatile-bearing regolith and fine dust. The fine dust response is comparatively lower at several of the higher excitation frequencies, despite exhibiting a lower median natural frequency.

The results demonstrate that capsule-level acceleration cannot necessarily be interpreted as equivalent to the acceleration experienced by material contained within the sample container. Consequently, two samples subjected to the same capsule-level vibration environment may exhibit different mechanical responses.

For the modeled excitation frequencies, the median responses at selected frequencies further illustrate this behavior. At 50 Hz, the median acceleration was approximately 0.053 g for bulk regolith, 0.014 g for fine dust, and 0.053 g for volatile-bearing regolith. At 200 Hz, the corresponding median accelerations decreased to approximately 0.0032, 0.00033, and 0.0010 g, respectively. These values represent modeled sample accelerations rather than measurements of returned lunar material.

The probabilistic spread is also substantial, particularly near frequencies where the assumed natural frequency distributions overlap with the excitation frequencies. For example, at 50 Hz, the 95th-percentile modeled acceleration reached approximately 0.24 g for bulk regolith, 0.28 g for fine dust, and 0.23 g for volatile-bearing regolith. This divergence between median and upper-percentile responses illustrates the importance of representing sample properties probabilistically rather than relying solely on a single nominal mechanical response.

The analysis provides a conceptual demonstration of how a vibration history measured at the capsule or sample container level could be coupled with a mechanical model to estimate the range of responses experienced by different sample materials. It does not establish a threshold for sample damage or science loss. Such interpretation would require experimentally constrained material properties, calibrated mechanical models, and experimentally determined disturbance or damage thresholds.

### 8.7. Maximum Modeled Sample Response

**Figure 7:**
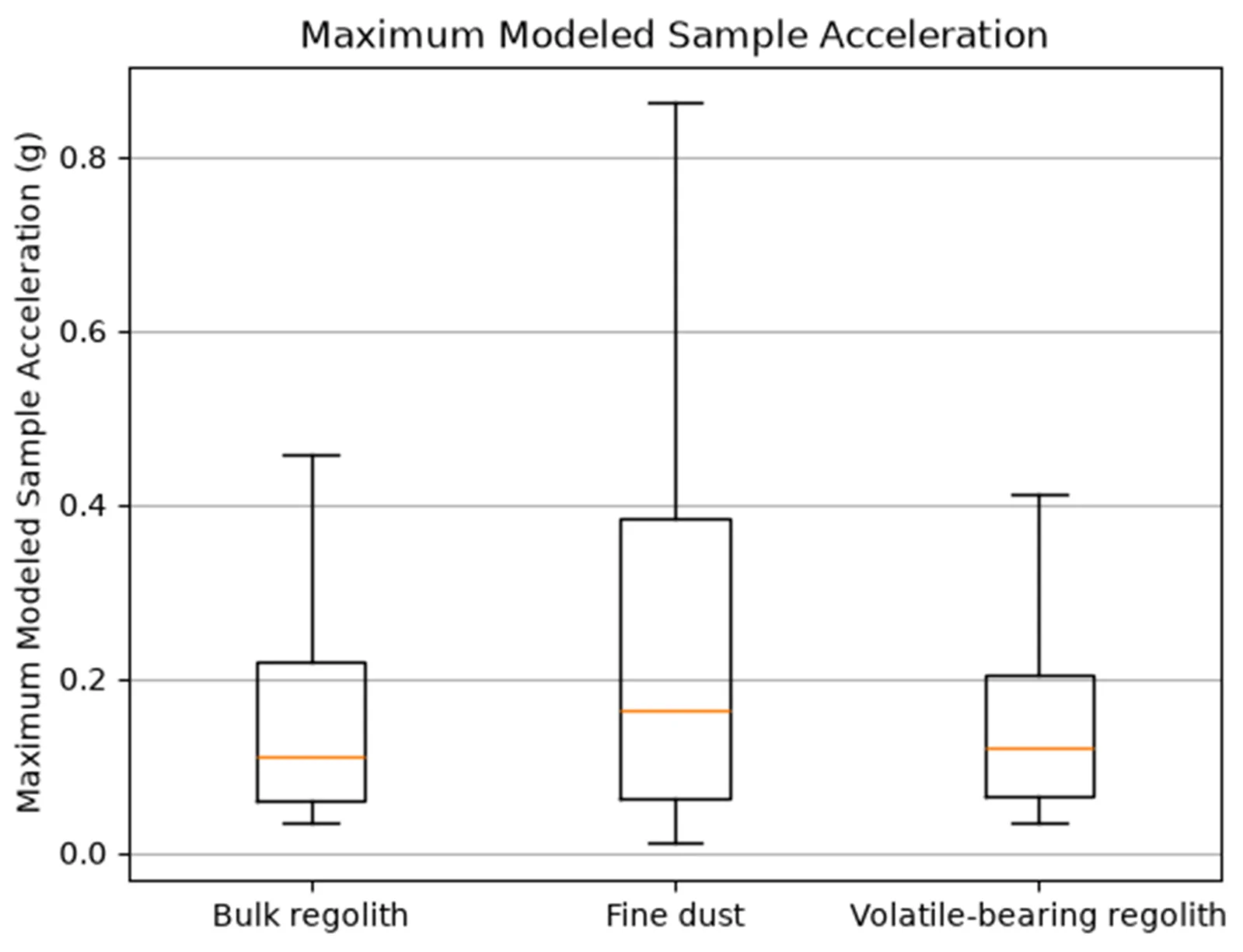
Maximum modeled sample acceleration across the frequency sweep for the three representative lunar sample archetypes. Fine dust exhibits the highest median maximum modeled acceleration and the broadest distribution, while bulk and volatile-bearing regolith show lower and comparatively narrower response distributions.

To compare the overall magnitude of the modeled response among the three sample archetypes, the maximum sample acceleration across the frequency sweep was determined for each Monte Carlo realization. This provides a single response metric for each realization while retaining the uncertainty associated with the assumed sample properties. The resulting distributions are shown as box-and-whisker plots in Figure 7. The bulk regolith archetype produced a median maximum acceleration of 0.110 g, with 5th and 95th percentile values of approximately 0.038 and 0.496 g, respectively. The fine dust archetype exhibited the largest median maximum acceleration, at 0.165 g, with a 5th–95th percentile range of approximately 0.016–0.723 g. The volatile-bearing regolith archetype produced an intermediate median value of 0.119 g, with 5th and 95th percentile values of approximately 0.037 and 0.366 g, respectively.

The modeled maximum responses also extended to substantially higher values for individual Monte Carlo realizations. The maximum acceleration obtained across the realizations was approximately 0.82 g for bulk regolith, 1.23 g for fine dust, and 0.62 g for volatile-bearing regolith. These individual maximum values represent the upper end of the modeled parameter space and should not be interpreted as expected acceleration levels for returned samples.

The frequency associated with the maximum response also differed among the archetypes. The median peak frequency was approximately 52.2 Hz for bulk regolith, 18.2 Hz for fine dust, and 40.3 Hz for volatile-bearing regolith. Thus, the archetypes differed not only in the magnitude of their maximum modeled responses, but also in the frequency range at which those responses were most likely to occur.

The larger modeled response of the fine dust archetype is notable because this archetype also exhibited the lowest median natural frequency. This result illustrates that the magnitude of the sample response cannot be inferred from natural frequency alone. The response depends on the interaction between the imposed vibration frequencies, the distribution of natural frequencies, and the assumed damping and mechanical properties of the sample.

The broad distributions shown in Figure 7 further emphasize the importance of uncertainty in the assumed material properties. In particular, the relatively large interquartile range and whisker extent for fine dust indicate that a wide range of maximum responses is possible within the parameter space explored by the model. This variability is a consequence of the assumed mechanical property distributions and should therefore be interpreted as model sensitivity rather than as an experimentally established range of lunar sample response.

## 9. Discussions

### 9.1. Linking Container-Level Vibration to Sample Response

A vibration measurement obtained at the sample container provides a direct record of the mechanical environment at the container boundary, but does not by itself establish the mechanical response of the material contained within it. Structural resonances, mounting conditions, damping, sample geometry, density, stiffness, and granular behavior can modify the vibration transmitted to the sample. The probabilistic model presented here provides a first-order analytical link among these quantities by propagating uncertainty in representative sample properties into natural frequency and acceleration response distributions. The results demonstrate that identical imposed vibration environments can produce substantially different predicted responses among lunar sample archetypes.

An important result of the analysis is that the archetype exhibiting the lowest median natural frequency did not necessarily exhibit the lowest maximum acceleration. Fine dust produced the highest median maximum modeled acceleration and the broadest response distribution, illustrating that susceptibility to a particular excitation environment cannot be inferred from natural frequency alone.

This framework should not be interpreted as a direct prediction of sample damage or volatile loss. Establishing such relationships would require progressive experimental and numerical validation, including measurements of representative material properties, mechanical modeling of the container and sample, and experimentally determined thresholds for scientifically relevant forms of sample disturbance. Finite-element methods could be used to characterize structural and container interface dynamics, while discrete-element approaches could provide greater fidelity for granular sample behavior. Experimental calibration using lunar regolith simulants or appropriate analog materials would further constrain the assumed mechanical property distributions. Ultimately, experimentally established disturbance thresholds could allow measured container-level vibration histories to be translated into quantitative assessments of potential sample alteration.

### 9.2. Limitations and Implications for Lunar Volatile Samples

The potential consequences of vibration for volatile-bearing lunar samples should be distinguished according to the level of available evidence. The present analysis does not directly demonstrate vibration-induced sublimation, phase changes, volatile redistribution, or other forms of volatile loss. Instead, it provides a probabilistic estimate of the mechanical response of representative lunar sample archetypes to an imposed vibration environment. Potential links between this mechanical response and volatile preservation remain hypotheses that require experimental validation.

Establishing quantitative relationships between measured container vibration and scientifically significant sample alteration would require experimentally determined material properties and disturbance thresholds, together with mechanical or particle-scale modeling where appropriate. The absence of such calibration data is an important limitation of the present framework, but it also identifies a practical opportunity for future sample return missions. Measurements of vibration at or near the sample container could provide a documented mechanical history that, when combined with experimentally constrained response models, would enable more quantitative assessment of potential sample disturbance. In this context, container-level vibration monitoring is best regarded as an enabling measurement for future sample integrity studies rather than, by itself, a diagnostic of sample alteration or science loss.

## 10. Conclusions

Planetary sample return missions expose returned materials to a range of mechanical environments from launch and cruise through entry, descent, landing, recovery, transport, and curation. Although vibration and shock have been characterized at several points along this mission chain, measurements at the sample container level remain comparatively less well documented. This distinction is important because the mechanical response of a contained sample cannot necessarily be inferred directly from the vibration measured elsewhere on the spacecraft or capsule.

To examine this relationship, we developed a probabilistic analytical framework for representative lunar sample archetypes using Apollo-derived sample container geometries and a 10,000-realization Monte Carlo model. Uncertainty in sample density, effective stiffness, and damping was propagated into natural frequency, transmissibility, and sample acceleration responses in a representative multi-frequency vibration environment. The results indicate that different lunar sample archetypes can exhibit substantially different dynamic responses to the same imposed vibration environment, with fine dust showing greater variability in modeled response than the bulk and volatile-bearing regolith archetypes.

These results do not establish vibration-induced volatile loss, sample damage, or other forms of scientific loss. Rather, they demonstrate that uncertainty in sample mechanical properties can produce significant uncertainty in the mechanical response of returned material. Establishing quantitative relationships between container-level vibration and scientifically relevant sample alteration will require further experimental characterization, calibrated mechanical models, and, where appropriate, particle-scale simulations and experimentally determined disturbance thresholds.

Within this context, vibration monitoring should be considered a potentially valuable component of future planetary sample return systems. Measurements at or near the sample container could preserve a record of the mechanical environment experienced by returned material, providing environmental context for post-return sample assessment. Such measurements would not independently determine the cause of subsequent sample changes, but could be combined with mechanical modeling and post-return analyses to evaluate whether mission-induced mechanical disturbance was a plausible contributor. For future lunar volatile sample return missions, documenting this mechanical history through experiments, DEM, and disturbance threshold testing may therefore provide an additional layer of information for evaluating sample integrity and interpreting potential scientific loss during transport and curation.

## Figures and Tables

**Table 1:** Summary of sensor types associated with historic planetary sample return missions, with sensor location and relevance to sample. The knowledge gap row illustrates that a dedicated vibration sensor is currently lacking.

Mission	Sensor Type	Sensor Location	Mission Phase	Relevance to Sample
Apollo	Seismic/acoustic	Ground	Launch/landing/reentry	Indirect
Genesis	--	Capsule	Landing/recovery	Sample disturbance demonstrated
Stardust	Seismic, infrasonic, optical	Ground	EDL	Indirect
Hayabusa	External	Spacecraft, ground	EDL	Indirect
Hayabusa2	External	Spacecraft, ground	EDL	Indirect
OSIRIS-Rex	Accelerometry, external	Spacecraft, ground	EDL	Indirect
Knowledge gap	Dedicated vibration sensor	Container	Collection to curation	Direct/near-direct

**Table 2:** Representative mechanical property distributions used in the Monte Carlo analysis.

Parameter	Bulk Regolith	Fine Dust	Volatile-Bearing Regolith	Basis	Distribution
Density *ρ*	1.30–1.90 g/cm^3^	1.10–1.60 g/cm^3^	1.30–1.90 g/cm^3^	Assumption	Uniform
Effective stiffness $k_{eff}$	100–100,000 N/m	10–10,000 N/m	30–30,000 N/m	Assumption	Log-uniform
Damping ratio ζ	0.03–0.07	0.02–0.05	0.04–0.10	Assumption	Uniform
Sample Mass	15 g	15 g	15 g	Nominal modeling assumption	Fixed

## Data Availability

All data used in this paper came from published sources listed in the references. The Monte Carlo data represented in the study are openly available in Monte Carlo Model for Vibration Sensor re: Apollo Sample Capsule—Version 1. Zenodo. https://doi.org/10.5281/zenodo.22234557.

## Abbreviations

The following abbreviations are used in this manuscript:

EDL	Entry/descent/landing
OSIRIS-REx	Origins, Spectral Interpretation, Resource Identification, and Security—Regolith Explorer
LEAG	Lunar Exploration Analysis Group
ExMAG	Extraterrestrial Materials Assessment Group
SAT	Special action team
SRC	Sample return capsule
ANGSA	Apollo next-generation sample analysis
SSRC	Stardust sample return capsule
UTTR	Utah Test and Training Range
IMU	Inertial measurement unit
MEMS	Microelectromechanical system
RICE	Roosevelt Island Climate Evolution
WAIS	West Antarctic Ice Sheet
PSR	Permanently shadowed regions
DEM	Discrete element modeling
FFT	Fast Fourier transform
PSD	Power spectral density

## References

[1] National Academies of Sciences, Engineering, and Medicine (2023). Origins, Worlds, and Life: A Decadal Strategy for Planetary Science and Astrobiology 2023–2032.

[2] Weber R.C., Lawrence S.J., Cohen B.A., Bleacher J.E., Boyce J.W., Collier M.R., Draper D., Fagan A.L., Fassett C.I., Gaddis L. The Artemis III science definition team report. Proceedings of the 52nd Lunar and Planetary Science Conference.

[3] Beaty D.W., Grady M.M., McSween H.Y., Sefton-Nash E., Carrier B.L., Altieri F., Amelin Y., Ammannito E., Anand M., Benning L.G. (2018). The Potential Science and Engineering Value of Samples Delivered to Earth by Mars Sample Return-Final Report.

[4] Grady M.M. (2020). Exploring Mars with returned samples. Space Sci. Rev..

[5] Lunar Exploration Analysis Group (LEAG), Extraterrestrial Materials Assessment Group (ExMAG) (2024). Special Action Team on Artemis Samples: Cold Conditioned and Volatile Samples. https://www.lpi.usra.edu/leag/reports/ASR-SAT_CCVAS_Report.pdf.

[6] Gershman R., Adams M., Mattingly R., Rohatgi N., Corliss J., Dillman R., Fragola J., Minarick J. (2004). Planetary protection for Mars sample return. Adv. Space Res..

[7] White T., Hwang H., Ellerby D., Gasch M., Beck R., Vander Kam J., Venkatapathy E. (2020). Thermal Protection System Materials for Sample Return Missions.

[8] Brownlee D.E., Tsou P., Anderson J.D., Hanner M.S., Newburn R.L., Sekanina Z., Clark B.C., Hörz F., Zolensky M.E., Kissel J. (2003). Stardust: Comet and interstellar dust sample return mission. J. Geophys. Res. Planets.

[9] Lauretta D., Balram-Knutson S., Beshore E., Boynton W., Drouet d’Aubigny C., DellaGiustina D.N., Enos H.L., Golish D.R., Hergenrother C.W., Howell E.S. (2017). OSIRIS-REx: Sample return from asteroid (101955) Bennu. Space Sci. Rev..

[10] Sansom E.K., Devillepoix H.A., Yamamoto M.Y., Abe S., Nozawa S., Towner M.C., Cupák M., Hiramatsu Y., Kawamura T., Fujita K. (2022). The scientific observation campaign of the Hayabusa-2 capsule re-entry. Publ. Astron. Soc. Jpn..

[11] Silber E.A., Bowman D.C., Albert S. (2023). A Review of Infrasound and Seismic Observations of Sample Return Capsules since the End of the Apollo Era in Anticipation of the OSIRIS-REx Arrival. Atmosphere.

[12] Donn W.L., Ewing M. (1972). Resonant coupling of ocean Rayleigh waves to atmospheric shock waves from Apollo rockets. J. Geophys. Res..

[13] Hilton D.A., Henderson H.R. (1974). Measurements of sonic-boom overpressures from Apollo space vehicles. J. Acoust. Soc. Am..

[14] Desai P.N., Cheatwood F.M. (2001). Entry dispersion analysis for the genesis sample return capsule. J. Spacecr. Rocket..

[15] Mitcheltree R.A., Wilmoth R.G., Cheatwood F.M., Brauckmann G.J., Greene F.A. (1999). Aerodynamics of Stardust sample return capsule. J. Spacecr. Rocket..

[16] Yamamoto M.Y., Ishihara Y., Hiramatsu Y., Kitamura K., Ueda M., Shiba Y., Furumoto M., Fujita K. (2011). Detection of acoustic/infrasonic/seismic waves generated by hypersonic re-entry of the HAYABUSA capsule and fragmented parts of the spacecraft. Publ. Astron. Soc. Jpn..

[17] Lauretta D.S., Connolly H.C., Aebersold J.E., Alexander C.M.O.D., Ballouz R.L., Barnes J.J. (2024). & OSIRIS-REx Sample Analysis Team Asteroid (101955) Bennu in the laboratory: Properties of the sample collected by OSIRIS-REx. Meteorit. Planet. Sci..

[18] Fernando B.A., Charalambous C., Schmerr N., Craig T.J., Wolf J., Lewis K., Sansom E.K., Saliby C., McCleary M., Inman J. (2025). Array-based seismic measurements of OSIRIS-REx’s re-entry. Seismol. Res. Lett..

[19] Bierhaus E.B., Clark B.C., Harris J.W., Payne K.S., Dubisher R.D., Wurts D.W., Hund R., Kuhns R., Linn T., Wood J. (2018). The OSIRIS-REx spacecraft and the touch-and-go sample acquisition mechanism (TAGSAM). Space Sci. Rev..

[20] Acar C., Shkel A.M. (2003). Experimental evaluation and comparative analysis of commercial variable-capacitance MEMS accelerometers. J. Micromech. Microeng..

[21] Ji S., Zhang C., Gao S., Lian A. (2024). A near-vertical well attitude measurement method with redundant accelerometers and MEMS IMU/Magnetometers. Appl. Sci..

[22] Fitzpatrick J.J., Voigt D.E., Fegyveresi J.M., Stevens N.T., Spencer M.K., Cole-Dai J., Alley R.B., Jardine G.E., Cravens E.D., Wilen L.A. (2014). Physical properties of the WAIS Divide ice core. J. Glaciol..

[23] Williams J.P., Greenhagen B.T., Paige D.A., Schorghofer N., Sefton-Nash E., Hayne P.O., Lucey P.G., Siegler M.A., Aye K.M. (2019). Seasonal polar temperatures on the Moon. J. Geophys. Res. Planets.

[24] Landis M.E., Hayne P.O., Williams J.P., Greenhagen B.T., Paige D.A. (2022). Spatial distribution and thermal diversity of surface volatile cold traps at the lunar poles. Planet. Sci. J..

[25] Ahrens C.J., Fastook J., Petro N.E. (2025). Diverse Lunar Polar Permanently Shadowed Regions and Environmental Metrics for Site Planning Decision-Making. Adv. Space Res..

[26] Blewett D.T., Cahill J., Chabot N., Greenhagen B., Hibbitts C., Klima R., Lawrence D., Mandt K., Jorge I.N., Patterson W. (2021). Mission to Characterize Volatiles in Old, Cold, Permanently Shadowed Regions on the Moon. Bull. Am. Astron. Soc..

[27] Rasera J.N., Salinas-Farran L.E., Starr S.O., Schein V., Lomax B., McDonald F., Cilliers J.J., Hadler K. (2025). Vibration-induced size segregation of lunar regolith and simulants for in situ resource utilisation (ISRU). Space Planet. Resour..

[28] Boyd I.D., Trumble K.A., Wright M.J. (2010). Modeling of Stardust entry at high altitude, Part 1: Flowfield analysis. J. Spacecr. Rocket..

[29] Ajluni T., Everett D., Linn T., Mink R., Willcockson W., Wood J. (2015). OSIRIS-REx, returning the asteroid sample. 2015 IEEE Aerospace Conference.

[30] Grinstead J., Jenniskens P., Cassell A., Albers J., Winter M. Airborne observation of the Hayabusa sample return capsule Re-entry. Proceedings of the 42nd AIAA Thermophysics Conference.

[31] Zolensky M., Nakamura-Messenger K., Fletcher L., See T. (2008). Curation, spacecraft recovery, and preliminary examination for the Stardust mission: A perspective from the curatorial facility. Meteorit. Planet. Sci..

[32] Burnett D.S., Barraclough B.L., Bennett R., Neugebauer M., Oldham L.P., Sasaki C., Sevilla D., Smith N., Stansbery E., Sweetnam D. (2003). The Genesis discovery mission: Return of solar matter to Earth. Space Sci. Rev..

